# Rectus sheath abscess after laparoscopic appendicectomy

**DOI:** 10.4103/0972-9941.33275

**Published:** 2007

**Authors:** Vishwanath Golash

**Affiliations:** Department of Surgery, Sultan Qaboos Hospital, P. O. Box 98, Salalah - 211, Sultanate of Oman

**Keywords:** Appendicetomy, laparoscopy, rectus sheath abscess

## Abstract

Port site wound infection, abdominal wall hematoma and intraabdominal abscess formation has been reported after laparoscopic appendicectomy. We describe here a rectus sheath abscess which occurred three weeks after the laparoscopic appendicectomy. It was most likely the result of secondary infection of the rectus sheath hematoma due to bleeding into the rectus sheath from damage to the inferior epigastric arteries or a direct tear of the rectus muscle. As far as we are aware this complication has not been reported after laparoscopic appendicectomy.

## INTRODUCTION

Laparoscopic appendicectomy per se does not increase the incidence of complications.[1] Abdominal wall vessel injuries may occur during trocar insertion in laparoscopy leading to rectus sheath hematoma although infection of hematoma is a rare occurrence. The usual presentation is chills and rigor, acute abdominal pain associated with palpable painful, firm, abdominal mass corresponding to the rectus sheath. The hematoma takes the shape according to its location in the rectus sheath; above the arcuate line it appears as unilateral spindle shape and below the arcuate line it appears spherical in shape. Signs of urinary bladder and peritoneal irritation with rebound tenderness and involuntary guarding may be present. Rarely, a rectus sheath hematoma/abscess may rupture into the peritoneum, causing peritonitis. Clinically, Fothergill sign and Carnett's sign help in differentiating it from intrabdominal pathology.[2] Ultrasound, computerized tomography (CT) scan and magnetic resonance imaging are accurate in localizing the abscess and excluding intraabdominal pathology.

## CASE REPORT

**Figure 1:**
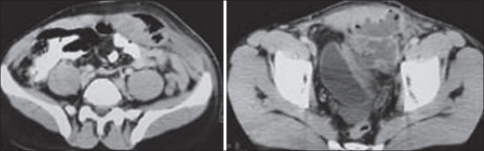
CT scan shows loculation with air pockets in the left lower rectus sheath and displacement of urinary bladder

**Figure 2:**
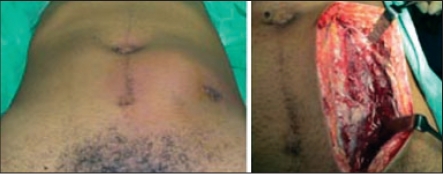
Patient picture showing spherical mass left lower abdomen, port sites of appendicetomy (left) and operative findings (right)

A 26-year-old male presented with the history of lower abdominal pain, fever, vomiting and increasing swelling over the lower abdomen for the last one week. He had laparoscopic appendicectomy elsewhere three weeks ago and was discharged home on the third postoperative day. He had been feeling unwell with lower abdominal pain since his discharge from the hospital and was given a week's course of antibiotics and analgesic in a private clinic. His abdominal examination revealed: the laparoscopic port site scar noticed at the umbilicus, left iliac fossa and the suprapubic area [Figure 1], generalized abdominal tenderness and guarding, visible and palpable spherical mass in the left side of abdomen occupying the left paraumbilical and suprapubic area with signs of inflammation. Laboratory tests showed leukocytosis and neutrophilia. Coagulation profile was within the normal range. An abdominal ultrasound revealed air fluid level in the left anterior abdominal wall with a cavity 9 × 5 cm in size suggestive of an abscess. The CT scan of the abdomen showed extraperitoneal collection, loculation with air pockets in the left lower rectus sheath, rectus muscle was infiltrated. The collection was displacing the urinary bladder with no intraperitoenal communication and no intraperitoneal fluid collection [Figure 2]. A diagnosis of rectus sheath abscess was made.

The rectus sheath abscess was opened by a long left paramedian incision. The rectus muscle and the inferior epigastric vessels were found macerated; 300ml of offensive smelling pus was evacuated. There was a deep abscess cavity extending behind the pubis symphysis but not communicating with the peritoneal cavity. The necrotic bits of rectus muscle were debrided. The wound was left open and secondary suturing was done after two weeks. Culture grew *Escheria coli*.

## DISCUSSION

Rectus sheath hematoma/abscess is a potentially serious condition with significant morbidity and mortality.[3] Most likely this patient had secondary infection of the rectus sheath hematoma leading to abscess formation. A hematoma within the rectus sheath occurs when the epigastric vessels or the rectus muscle are lacerated during trocar insertion. A large amount of blood or pus can accumulate within the rectus sheath and would only be noticed once the swelling becomes obvious. Trauma to abdominal wall blood vessels has been reported in up to 3.4% of laparoscopic procedures.[4] Most commonly injured vessels in laparoscopic procedures are the epigastric vessels. The epigastric vessels are usually located in the area between 4 and 8 cm from the midline. Staying away from this area, either medially or laterally, will determine the safety zone of entry the abdominal wall.[5] Proper understanding of the abdominal wall anatomy is essential in avoiding the injury to vessels.
